# Supplementary material for: Food safety practices of individuals before and after the emergence of COVID-19: A pre- and post-comparative analysis
Source: PLoS One. 2025 Apr 29;20(4):e0322235. doi: 10.1371/journal.pone.0322235 (PMC12040163; doi:10.1371/journal.pone.0322235)
Supplement: Attachment — Submitted filename: Response to Reviewers.pdf [file pone.0322235.s003.pdf]

Reviewer 1 Comments

Dear reviewer, we appreciate your commitment to review our manuscript entitled “Food safety practices of individuals before and after the emergence of COVID-19: a pre and post comparative analysis”. Your comments are valuable and can improve the quality of our manuscript. We forwarded a point-by-point response for each of your comments as shown below. All the revisions are showed in Microsoft track change of the revised manuscript.

Comment #1: much volume of result narration at the abstract section, better to reduce

Response #1: *The comment is accepted. The result section is edited. The detail is indicated in Microsoft track change.*

Comment #2: From January 2020 to December 2023 is only three years not four years

Response #2: T*he number of years is corrected.*

Comment #3: Better to include non-response rate in the methodology section

Response #3: *It is a constructive comment. The nonresponse rate is included and elaborated in the methods section.*

Comment #4: How the 10th food buyer in each open market is selected?

Response #4: *Thanks for the comment. The information about how 10th food buyer was selected was described in the method section. For your reference, I have copy-pasted the sentences from the method section here and marked red in the methods section.*

“There were two data collectors working together during the data collection. One of them conducted the survey and the other had maintained the sequence of participants. The sequence of the participants was based on the queue that the food buyers make to the casher/shop clerk. From the queue, every 10th food buyer who volunteered to participate and was over 18 years old were surveyed.”

Comment #5: Operationally define what 'open market' is.

Response #5: *Sorry for the confusion occurred. We have edited the phrase ‘open market’ to ‘open-air market’ and defined it in the context of this study. The detail is indicated in the Microsoft track change of the revised manuscript.*

Comment #6: How can ' Language" be a barrier in study? Why not translate to different local languages?

Response #6: *Dear reviewer, thanks for the comment. As you said it is possible to translate the survey question to different languages, but as we explained in our manuscript, we do not have a research team member that can speak other local languages other than Amharic. To employ data collectors that can speak different local languages, we had budget constraints. Since our survey is not self-administered, face to face communication between the surveyor and the participants was necessary and we consider language as the most significant barrier in our study. We mentioned this issue in the limitation section of our study.*

Comment #7: There are sample size fallacies as 385 in the methodology part and 396 in the result section.

Response #7: *It is corrected in the revised manuscript.*
