# Supplementary material for: Food safety practices of individuals before and after the emergence of COVID-19: A pre- and post-comparative analysis
Source: PLoS One. 2025 Apr 29;20(4):e0322235. doi: 10.1371/journal.pone.0322235 (PMC12040163; doi:10.1371/journal.pone.0322235)
Supplement: Attachment — Submitted filename: Response to Reviewers (Revised).pdf [file pone.0322235.s004.pdf]

Response to reviewer comments

Reviewer 2 Comments

Dear reviewer and editor, we appreciate your commitment to reviewing our manuscript entitled “Food safety practices of individuals before and after the emergence of COVID-19: a pre and post comparative analysis”. Your comments are valuable and can improve the quality of our manuscript. We forwarded a point-by-point response to your comments, as shown below. All the revisions are shown in Microsoft track change of the revised manuscript.

Comment #1: Clarify some of the more technical statistical terms for non-expert readers, such as "Wald X^2" and "dummy variables." Brief explanations could help make the analysis more accessible to a wider audience.

Response #1: Thanks for this comment. The above-mentioned statistical terms are described in the data analysis sub-section of the revised manuscript. We hope that the terms are now understandable to readers.

Comment #2: Simplify some of the longer sentences and correct minor punctuation errors to enhance clarity. Ensure consistency in formatting numerical data and percentages.

Response #2: Some long sentences in the manuscript have been shortened, and the punctuation marks have been revised and corrected. The number and percentage font sizes in tables have been edited to minimize the size, and they are now consistent throughout the manuscript.

Comment #3: Consider expanding on how your findings can be integrated into national food safety policies or education programs. This could strengthen the practical implications of your research.

Response #3: Thanks for this valuable comment. The implications of our findings in respect to the national food safety standards and education and research programs are included in the conclusion section.
