# Supplementary material for: Food safety practices of individuals before and after the emergence of COVID-19: A pre- and post-comparative analysis
Source: PLoS One. 2025 Apr 29;20(4):e0322235. doi: 10.1371/journal.pone.0322235 (PMC12040163; doi:10.1371/journal.pone.0322235)
Supplement: Attachment — Submitted filename: Response_to_Reviewers_auresp_3.pdf [file pone.0322235.s005.pdf]

Authors' Response: PONE-D-24-01799R2

To the Editor,

First and foremost, we would like to express our sincere gratitude to both the Editor and the reviewer for their valuable time and effort in reviewing our research paper titled *"Food safety practices of individuals before and after the emergence of COVID-19: a pre- and post-comparative analysis."* We have carefully considered all the comments provided by the editor and have made explicit revisions to address each one (detailed below). The modifications are highlighted in red in the revised manuscript.

Editor's comments

This study explores "Food safety practices of individuals before and after the emergence of COVID-19: a pre- and post-comparative analysis the authors have done a commendable job, and I congratulate them. I have some critical comments and some questions for the authors, which, if addressed, will increase the readability and understandability of the manuscript. I have put it section by section for ease of communication

***Response**: We appreciate your encouragement, and all the comments provided have been found constructive and addressed individually as follows.*

Methods:

1. Please include the study period (the same comments need to be incorporated in the section of methods also)

***Response**: The study period is added in both abstract and methods sections.*

2. Line 19: You have mentioned …." three regional (Dessie, Kombolcha, and Debre Birhan) cities of Ethiopia" … it is crystal clear these are a pocket area in Amhara reginal state. Thus, you have state this as (Amhara Reginal State (Dessie, Kombolcha, and Debre Birhan)

***Response**: The comment is accepted and the study cities are stated as suggested.*

3. Line 21: you have stated that …" An equal number of participants were surveyed from metropolitan and regional cities" …. why an equal number? Have you assured the representativeness of the sample? How you choose the food shops?

***Response**: Since this study aimed to examine the food safety impacts of COVID-19 in both metropolitan and regional cities, an equal number of samples was targeted for collection. To minimize the influence of sample size variations on the statistical significance of the study, equal sample quotas were allocated to both the metropolitan city and regional cities. This is elaborated in the methods section in detail.*

4. Which software used for data entry and analysis should be indicated and when you declare statistical significance should be clearly mentioned

***Response**: IBM SPSS Version 28 was used and it is now more elaborated in the abstract section in some parts of the methods section.*

5. Result: change this to <Results >

***Response**: The comment is accepted and "Result" is changed to "Results"*

6. Conclusion: should be <conclusions > and the conclusion should be based only on the current study findings, according to your study objective. Don't put general ideas and common sense here. All the recommendations should align with your study findings.

***Response**: The conclusion has been updated to "Conclusions," and the section has been revised based on the findings of this research. The changes have been highlighted in red in the revised manuscript.*

Conceptual model:

1 Line 61: Fig 1: “Concept map of COVID-19’s impact on food safety and its indirect impact on health based on the findings of previous studies” ……have a citation as you have taken from finding of previous studies

***Response**: Citations added.*

MATERIALS AND METHODS:

1. Generally. Reporting guidelines: Please ensure you follow the appropriate reporting guidelines when preparing your manuscript and submit the completed checklist as supplementary material. Please state in the methods which guidelines were consulted when preparing the manuscript. More information on reporting guidelines can be found on the EQUATOR website

***Response**: As recommended by scholars, the Strengthening Reporting of Observational Studies in Epidemiology (STROBE) reporting guideline was applied to research designs similar to ours, specifically the retrospective cross-sectional design. The details are explained in lines 122-124 of the revised manuscript, and a checklist is provided as supporting information.*

2. Study design, sample size, and sampling procedure: The study seems that community type of study, if that is case why don’t use a design effect in calculating the sample size?

***Response**: Thank you for your constructive comment. Our sampling strategy was simple random sampling rather than a complex method such as cluster or stratified sampling. Since the design effect is typically recommended for complex sampling techniques and we believed it is not mandatory for our sampling approach, we did not used it for our sample size calculation.*

3. If you said that, individuals were selected using random sampling methods… How you create the sampling frame both for individuals and included open air market

***Response**: Creating a sampling frame in an open-air market with a large population was not possible. Therefore, only individuals in the queue at the billing area were considered, using the queue as the sampling frame for such a large gathering. This has been elaborated in the revised manuscript, with the following paragraph among the information included in the methods section.*

“Since participants were chosen from the existing queue leading to the billing area, individuals outside this queue were not allowed to take part. Given the large number of people in an open-air market, creating a complete sampling frame was impractical. Following the recommendation of Mooney and Garber (14), the queue itself was used as the sampling frame in such large population size.”

4. Line 119-121: you have mentioned that …” As the data collection was not self-administered and was conducted in face-to-face interviews by trained data collectors, ultimately there were no non-respondent participants in the survey. The researchers had expected a 3% non-response rate and therefore the total sample collected, including the expected nonresponse rate” ……I have afraid of non-response rate of 3%, you chose? what if an individual’s withdraw in middle of data collection? once agreed to fill and refuse to participate? what if selected individuals absent from the works? what if he/she overloaded with certain over job? How you tackled these factors?

***Response**: A detailed elaboration is provided in the methods section of the revised manuscript. If a participant changes their mind during the survey or refuses to participate from the outset, the data collectors proceed to the next participant. Thanks for the comment and the confusing sentence is removed from the revised manuscript.*

5. Please include both “study variables” and “operational definitions of terms “as different sub titles

***Response**: Thank you for your comments. The sections on “Study Variables” and “Operational Definitions of Terms” have been incorporated into the revised manuscript, spanning lines 147–197 of the Methods section.*

6. Ethical approval: Personally, I think the study conducted in Ethiopia and your target (study)populations are Ethiopian. But you have mentioned that you have obtained "Ethics approval from the University of New England Human Research Ethics Committee 133 (HREC) with an approval number: HE22-173" … needs a further elaboration as you had to have an approval also from National ethics review Board of Ethiopia

***Response**: Apologies for not mentioning the ethical approval obtained from the first author's home institution, Wollo University, Ethiopia. Wollo University endorsed the ethical clearance granted by the University of New England, and we received an exemption letter for this, which is attached as supporting file.*

Results

1. Please have a "Subtitle "which clearly enhance the quality of your manuscript.

***Response**: Subtitles are added in the results section of the revised manuscript.*

2. You have to report with Adjusted Odd Ratio

***Response**: Thank you for your comment. The analysis was conducted using the adjusted odds ratio; however, the report initially presented it as the crude odds ratio. This has now been corrected to reflect the adjusted odds ratio.*

3. The results section needs tightening. For the descriptive section, either you report results as percentages or proportions (e.g., n/N). The way you have it now is confusing

***Response**: The result is corrected as per the comment.*

Discussion

1. Generally, the discussion lacks depth, as it only compares the results across studies. When comparing the study findings with reports from the existing literature, it is recommended to comment on the reasons for the observed discrepancy based on the theoretical framework or give a plausible explanation. It is also required to indicate its clinical and theoretical implications on the topic of interest.

***Response**: Detailed reasoning has been incorporated into the discussion section of the revised manuscript, with the changes highlighted in red.*

2. Further elaboration on statistical assumptions and policy implications would enhance transparency.

***Response**: A new subheading for policy implications has been added under the discussion section, and the statistical assumptions have been further elaborated and highlighted in red.*

Strengths and Limitations of Study

1. Limitations should address potential biases (e.g., selection or recall bias), constraints in data collection (e.g., self-reporting or incomplete data), and generalizability of the findings to other populations or settings.

***Response**: The limitation section is revised as per the comment.*

Conclusions and other section

1. The conclusion should be based only on the current study findings, according to your study objective. Don't put general ideas and common sense here. All the recommendations should align with your study findings.

***Response**: The conclusion section is revised as per the comment and the changes are marked red.*

2. Finally, don’t forget attaching the most important section at end of your manuscript such as Data Sharing Statement, Ethical Approval, Acknowledgments, Authors’ Contributions, Funding Statement and Disclosure

***Response**: The suggested important sections are added under the newly included declaration section.*
