# Supplementary material for: Food safety practices of individuals before and after the emergence of COVID-19: A pre- and post-comparative analysis
Source: PLoS One. 2025 Apr 29;20(4):e0322235. doi: 10.1371/journal.pone.0322235 (PMC12040163; doi:10.1371/journal.pone.0322235)
Supplement: Attachment — Submitted filename: Response_to_Reviewers_auresp_4.pdf [file pone.0322235.s006.pdf]

Authors' Response: PONE-D-24-01799R3

To the Editor,

First and foremost, we sincerely thank the Editor for dedicating valuable time and effort to review our research paper entitled, "*Food safety practices of individuals before and after the emergence of COVID-19: a pre- and post-comparative analysis.*" We have thoroughly reviewed all the comments provided and have made specific revisions to address each point (outlined below). The changes have been marked in red in the revised manuscript.

Journal requirements:

Please review your reference list to ensure that it is complete and correct. If you have cited papers that have been retracted, please include the rationale for doing so in the manuscript text, or remove these references and replace them with relevant current references. Any changes to the reference list should be mentioned in the rebuttal letter that accompanies your revised manuscript. If you need to cite a retracted article, indicate the article's retracted status in the References list and also include a citation and full reference for the retraction notice.

***Response**: To the best of our knowledge, no retracted references have been used in the manuscript. The EndNote reference manager, utilizing the Vancouver citation style, was used for reference and citation arrangements.*

Editor's comments

Methods:

1. Inconsistency with your study period (line 20: 16 April 2023, and 31 June 2023; line 122: 6 April 2023 to 30 June 2023) please check it

***Response**: We apologize for the typographical errors and confirm that they have been corrected in the revised manuscript.*

2. Line 27 "The data was entered IBM SPSS version 28 and analyzed using ordinal logistic regression" …. how that? if you entered the data using SPSS, how you control logical skipping and others. Why don't you used EPI data software for data entry? and moreover, once you amended the stated statement, move it to methods section of abstract.

***Response**: Thank you for this valuable comment. We have included the following paragraph to enhance clarity for readers. To minimize inconsistencies related to data formatting and values, we utilized IBM SPSS software for both data entry and statistical analysis. The use of this software is also briefly mentioned in the methods section of the abstract.*

"After data entry, data cleaning and management procedures were performed using SPSS Syntax commands in preparation for statistical analysis. These commands are particularly effective for identifying and correcting errors or inconsistencies related to data formatting and values, including missing data, skips, misinterpretations, duplicates, and typographical errors."

MATERIALS AND METHODS:

1. Explicitly, you have employed STROBE Statement—Checklist of items as per comments but some of the points needs to be addressed in the details and you need to have a subtitle such for instance "Bias" …. Like recall bias, social desirability bias and how you mitigate it

***Response**: Thank you for your feedback. To resolve this issue, a new subheading has been added and further explained in the methods section of the revised manuscript.*

2. Line 147-158: what you mentioned under the subtitle of “study variables” need to move to section of “operational definition “. Rather here you are expected your dependent (outcome variables) and independent variables

***Response**: The indicated paragraphs are moved to the operational definition subsection.*

3. Line 160-188: Please re write section as “operational definitions of terms and measurement”. First define the listed term such as cleaning, separating and cooking, chilling etc. …. Then tell us how you measure it.

***Response**: The operational definition has been included, and the measurement method has been described as requested and highlighted in red in the revised manuscript.*
